# Blood - brain barrier breakdown and neurovascular unit failure in the progression of Alzheimer's disease

**DOI:** 10.6026/973206300221673

**Published:** 2026-03-31

**Authors:** Suvarna Prasad, Sangeeta Gupta, Asha Premlata Omega Oraon, Ana Paula Molina Recalde, Jessica Julieta Cañarte Moreira, Génesis Nathalie Moyano Salazar, Paolo David Torres Cañarte, Camila Inés Racines Navas

**Affiliations:** 1 Department of Biochemistry, AIIMS, Deoghar, Jharkhand, India; 2 Department of Physiology, AIIMS, Gorakhpur, Gorakhpur, Uttar Pradesh, India; 3 Department of Medicine, Universidad San Francisco de Quito, Ecuador; 4 Department of Medicine, Centro Oncológico del Norte, Antofagasta, Chile

**Keywords:** Blood-brain barrier (BBB), neurovascular unit (NVU), Alzheimer's disease (AD), cognitive impairment, cerebral perfusion

## Abstract

Alzheimer's disease (AD) is a progressive neurodegenerative disorder traditionally defined by amyloid-β deposition and tau pathology. Recent research highlights the role of blood-brain barrier (BBB) breakdown and neurovascular unit (NVU) dysfunction in disease initiation and progression. These factors contribute to neuroinflammation, cerebral hypoperfusion and impaired waste clearance. Despite this, the relationship between BBB integrity, NVU function and cognitive decline in AD remains inadequately understood. Data shows that blood-brain barrier disruption and neurovascular unit dysfunction are critical predictors of cognitive decline in Alzheimer's disease.

## Background:

Alzheimer's disease (AD) is the most common cause of dementia worldwide and represents a growing public health challenge due to aging populations and the absence of curative therapies. Clinically, AD is characterized by progressive memory impairment, cognitive decline and behavioral disturbances that ultimately lead to complete functional dependence. For decades, research on AD pathogenesis has been dominated by the amyloid cascade and tau hypotheses, which focus on the accumulation of amyloid-β plaques and neurofibrillary tangles as the primary drivers of neuronal degeneration [1]. Although these hallmarks remain central to disease pathology, increasing evidence suggests that AD is a multifactorial disorder involving not only neurons but also vascular, glial and immune components of the brain. Among these, dysfunction of the blood-brain barrier (BBB) and failure of the neurovascular unit (NVU) have emerged as critical contributors to disease initiation and progression [2]. The BBB is a highly specialized, selectively permeable interface that separates the circulating blood from the brain parenchyma. It is primarily formed by brain microvascular endothelial cells connected by tight junctions, supported by pericytes, astrocytic end-feet, basement membrane components, neurons and microglia [3]. Together, these elements constitute the neurovascular unit, which tightly regulates cerebral blood flow, nutrient transport, waste clearance and immune surveillance. Under physiological conditions, the BBB maintains brain homeostasis by restricting the entry of potentially neurotoxic substances, pathogens and peripheral immune cells, while allowing the controlled passage of essential molecules such as glucose, amino acids and oxygen. Disruption of this finely tuned system can have profound consequences for neuronal health and cognitive function [4]. Growing experimental, imaging and post-mortem evidence indicates that BBB breakdown occurs early in the course of AD, often preceding overt neuronal loss and classical neuropathological changes. Increased permeability of the BBB allows plasma proteins, inflammatory mediators and immune cells to enter the brain, triggering neuroinflammation, oxidative stress and synaptic dysfunction [5].

Leakage of blood-derived components such as fibrinogen, thrombin and albumin has been shown to activate microglia and astrocytes, leading to chronic inflammatory responses that exacerbate neuronal injury. These vascular insults may act synergistically with amyloid-β and tau pathology, amplifying neurodegenerative cascades rather than serving as mere downstream consequences of neuronal death [6]. Neurovascular unit failure in AD extends beyond simple barrier leakage and encompasses a broad spectrum of functional impairments. Endothelial dysfunction reduces nitric oxide availability and disrupts cerebral autoregulation, resulting in impaired neurovascular coupling and reduced cerebral blood flow [7]. Pericyte loss or degeneration, frequently observed in aging and AD brains, further destabilizes capillary integrity and compromises BBB maintenance. Astrocytes, which normally regulate ion balance, neurotransmitter recycling and vascular tone, undergo reactive changes that alter their supportive roles and contribute to metabolic dysregulation. Collectively, these alterations lead to chronic cerebral hypoperfusion, energy deficits and impaired clearance of metabolic waste products, including amyloid-β, thereby accelerating disease progression [8]. The interaction between BBB dysfunction and amyloid pathology represents a particularly important aspect of AD pathogenesis. Under normal conditions, amyloid-β is continuously cleared from the brain through multiple pathways, including enzymatic degradation, transport across the BBB and perivascular drainage. BBB breakdown disrupts receptor-mediated transport systems responsible for amyloid efflux while enhancing influx from the circulation, leading to net accumulation within the brain [9]. Furthermore, vascular amyloid deposition in cerebral vessels, known as cerebral amyloid angiopathy, directly damages endothelial cells and smooth muscle layers, creating a vicious cycle of vascular injury and amyloid accumulation. This bidirectional relationship underscores the central role of the neurovascular unit in maintaining amyloid homeostasis [10]. Inflammation and immune dysregulation also link BBB breakdown to neurodegeneration in AD. A compromised barrier permits peripheral immune cells and cytokines to access the brain, altering the normally immune-privileged environment of the central nervous system. This heightened inflammatory milieu promotes synaptic pruning, neuronal dysfunction and ultimately cell death [11]. Importantly, such vascular-immune interactions may help explain why systemic vascular risk factors, including hypertension, diabetes mellitus, hyperlipidemia and obesity, significantly increase the risk of developing AD. These conditions are known to impair vascular health and BBB integrity, suggesting that cerebrovascular dysfunction may serve as a mechanistic bridge between systemic disease and neurodegeneration [12]. Recognizing BBB and neurovascular unit dysfunction as integral components of AD has important clinical and therapeutic implications. Vascular changes may represent early biomarkers of disease, detectable before irreversible neuronal damage occurs. Advanced neuroimaging techniques and circulating biomarkers related to BBB integrity are increasingly being explored for early diagnosis and risk stratification [13]. Moreover, targeting the neurovascular unit offers novel therapeutic avenues that complement traditional anti-amyloid and anti-tau strategies. Interventions aimed at restoring endothelial function, stabilizing pericytes, reducing inflammation and improving cerebral perfusion may slow or prevent cognitive decline by preserving brain homeostasis [14]. Therefore, it is of interest to describe the critical role of blood-brain barrier dysfunction and neurovascular unit failure in Alzheimer's disease, as these factors may initiate and accelerate disease progression through mechanisms involving inflammation, hypoperfusion, and impaired waste clearance.

## Methodology:

## Study design and setting:

This original research was designed as an observational, cross-sectional analytical study conducted at a tertiary care teaching hospital with facilities for neurology, neuroimaging and laboratory biomarker analysis. The study aimed to evaluate blood-brain barrier (BBB) breakdown and neurovascular unit (NVU) dysfunction in patients with Alzheimer's disease and cognitively normal controls.

## Study population and sample size:

A total sample size of 100 participants was included in the study. The sample size was calculated based on previous literature reporting moderate effect sizes for BBB permeability differences between Alzheimer's disease patients and controls, with a confidence level of 95% and a power of 80%. Participants were divided into two groups:

[1] Group A (n = 50): Patients diagnosed with Alzheimer's disease

[2] Group B (n = 50): Age- and sex-matched cognitively normal controls

This equal allocation allowed adequate comparison between groups while minimizing selection bias.

## Inclusion criteria:

[1] Adults aged ≥60 years

[2] For Group A: Clinically diagnosed Alzheimer's disease based on standardized diagnostic criteria and cognitive assessment scores

[3] For Group B: No history of cognitive impairment, normal cognitive screening scores

[4] Willingness to provide informed consent (by participant or legally authorized representative)

## Exclusion criteria:

[1] History of stroke, traumatic brain injury, or other neurodegenerative disorders

[2] Active central nervous system infections or inflammatory diseases

[3] Severe systemic illness (*e.g.*, advanced renal, hepatic, or cardiac disease)

[4] Current use of medications known to significantly affect BBB permeability

[5] Contraindications to neuroimaging or blood sampling

## Data collection procedures:

## Clinical and cognitive assessment:

All participants underwent detailed clinical evaluation, including demographic data (age, sex, education), medical history and vascular risk factors such as hypertension, diabetes mellitus and dyslipidemia. Cognitive function was assessed using standardized neuropsychological tools appropriate for Alzheimer's disease staging. Disease severity in Group A was categorized into mild, moderate and severe stages based on cognitive scores.

## Assessment of blood-brain barrier integrity:

BBB integrity was evaluated using a combination of biochemical and imaging-based markers. Venous blood samples were collected under aseptic conditions to measure circulating biomarkers associated with BBB disruption and endothelial dysfunction. In a subset or where feasible, neuroimaging parameters indicative of BBB permeability and cerebral perfusion were assessed using standardized protocols. All measurements were performed by trained personnel blinded to group allocation.

## Evaluation of neurovascular unit function:

Markers reflecting NVU components including endothelial cells, astrocytes and pericyte-related dysfunction were analyzed using laboratory assays and imaging-derived indices. These parameters were correlated with cognitive performance and disease severity to assess the relationship between NVU failure and clinical progression of Alzheimer's disease.

## Ethical considerations:

The study protocol was reviewed and approved by the Institutional Ethics Committee. Written informed consent was obtained from all participants or their legally authorized representatives prior to enrollment. Confidentiality of participant data was strictly maintained and all procedures adhered to the ethical principles outlined in the Declaration of Helsinki.

## Statistical analysis:

Data were entered and analyzed using statistical software. Continuous variables were expressed as mean ± standard deviation or median with interquartile range, while categorical variables were presented as frequencies and percentages. Comparisons between groups were performed using independent t-tests or Mann-Whitney U tests for continuous variables and chi-square tests for categorical variables. Correlation and regression analyses were conducted to explore associations between BBB/NVU markers and cognitive scores. A p-value of <0.05 was considered statistically significant. This methodology ensured a systematic and reliable evaluation of BBB breakdown and neurovascular unit dysfunction in Alzheimer's disease within a well-defined sample of 100 participants.

## Results:

A total of 100 participants were included in the final analysis, comprising 50 patients with Alzheimer's disease (AD) and 50 cognitively normal controls. All enrolled participants completed clinical, cognitive, biochemical and imaging assessments and no data were missing for the primary outcome measures. The demographic and clinical characteristics of the study population are summarized in (Table 1 - see PDF). There were no statistically significant differences between the AD and control groups with respect to age, sex distribution, or years of education (p > 0.05). However, vascular risk factors such as hypertension and diabetes mellitus were significantly more prevalent in the AD group compared with controls (p < 0.05). As shown in Table 1 (see PDF), the higher burden of vascular comorbidities in the AD group supports the role of cerebrovascular dysfunction in disease pathogenesis. Cognitive assessment revealed significantly lower scores in the AD group compared with controls. The mean global cognitive score was markedly reduced among AD patients, with progressive decline observed across disease severity stages (Table 2 - see PDF). These findings confirm significant cognitive impairment in the AD group and validate the clinical stratification used in subsequent analyses. Markers of BBB integrity differed significantly between groups. AD patients demonstrated elevated circulating levels of BBB disruption markers compared with controls. These differences remained statistically significant after adjustment for age and vascular risk factors. As presented in (Table 3 - see PDF), BBB permeability was significantly increased in AD patients, indicating early and persistent barrier breakdown. Neuroimaging-based parameters reflecting NVU function showed significant impairment in the AD group. Reduced cerebral perfusion indices and altered neurovascular coupling were observed among AD patients compared with controls. These results, shown in Table 4 (see PDF), demonstrate significant NVU failure in Alzheimer's disease. A multivariate linear regression analysis was performed using STATA to evaluate the association between BBB disruption and cognitive decline after adjusting for potential confounders. The STATA-derived regression findings are summarized in (Table 5 - see PDF). The STATA regression model was statistically significant (F = 18.7, p < 0.001; R^2^ = 0.54), indicating that BBB disruption and NVU dysfunction independently predicted lower cognitive scores. As shown in (Table 5 - see PDF), increased BBB permeability was the strongest negative predictor of cognitive performance. Overall, the results demonstrate that Alzheimer's disease is associated with significant BBB breakdown and neurovascular unit failure, which is strongly correlated with cognitive impairment and disease severity (Tables 1-5 - see PDF).

## Discussion:

The findings of the present study demonstrate significant blood-brain barrier (BBB) breakdown and neurovascular unit (NVU) dysfunction in Alzheimer's disease (AD) patients compared with cognitively normal controls; supporting the growing paradigm that cerebrovascular dysfunction is integral to AD pathogenesis. These results are consistent with previous human studies that have implicated BBB disruption as an early pathological feature of cognitive decline and AD. Lin *et al.* (2021) [15] conducted a cross-sectional study measuring BBB permeability in mild cognitive impairment (MCI) compared to controls. They found increased permeability to small molecules in MCI patients, correlated with AD biomarkers, while large-molecule permeability was more linked to vascular risk factors. This suggests that BBB breakdown occurs early in the AD continuum and exerts differing effects depending on molecular size and underlying pathology. These observations align with our findings of increased BBB permeability markers in AD patients, reinforcing that barrier integrity impairment precedes and potentially contributes to cognitive dysfunction. Kurz *et al.* (2022) [16] performed a systematic review of human studies on BBB dysfunction in AD, reporting that structural changes, reduced transport protein expression, pericyte loss and proinflammatory mediators characterize barrier damage in AD brains. BBB disruption was detectable via imaging in early stages of disease and was potentiated by small vessel disease and ApoE genotype. The human evidence in their review mirrors our results showing significant NVU dysfunction and emphasizes the interaction between vascular risk factors and AD pathology. Huang *et al.* (2020) [17] reviewed the role of BBB integrity in AD and highlighted how NVU component interactions maintain homeostasis and how their dysfunction accelerates neurodegeneration. They underscored endothelial, pericyte and glial cell contributions to barrier breakdown and the consequent promotion of amyloid and tau pathology, which parallels our NVU imaging findings of reduced perfusion indices and altered coupling in AD subjects. Thus, consistent with our methodology and results, the breakdown of BBB integrity in AD likely results from complex interactions among NVU cells, not isolated endothelial failure. Chakraborty *et al.* (2017) [18] reviewed vascular contributions to AD, focusing on the loss of BBB protective function leading to impaired amyloid clearance and hypoperfusion.

Their narrative emphasized the relationship between vascular risk factors, cerebral blood flow deficits and cognitive decline. This conceptual framework supports the current study's observation of increased vascular burden (*e.g.*, hypertension and diabetes) in the AD group and suggests that systemic vascular health may exacerbate BBB breakdown and neurodegeneration. Zenaro *et al.* (2017) [19] described the BBB within the neurovascular unit and the role of immune cell trafficking in perpetuating barrier dysfunction and inflammation in AD. They highlighted how circulating leukocytes can migrate through activated endothelium, contributing to NVU disruption and further neurodegenerative processes. This mechanistic insight complements our findings of elevated inflammatory biomarkers and supports the concept that BBB breakdown is both a cause and consequence of AD-associated neuroinflammation. Collectively, these studies converge on the notion that BBB breakdown and NVU failure are not mere epiphenomena of Alzheimer's pathology but are pivotal contributors to disease progression. Our data extend this body of work by quantitatively demonstrating significant associations between barrier dysfunction, neurovascular impairment and cognitive decline within a well-characterized clinical cohort. The concordance between our findings and previous research underscores the reproducibility and clinical relevance of cerebrovascular markers as potential early indicators of AD risk and progression. However, differences in study designs should be acknowledged. While Lin *et al.* (2021) [15] utilized CSF albumin ratios and advanced imaging to assess small versus large molecule BBB permeability, our study relied on circulating BBB biomarkers and perfusion indices, which may reflect complementary but distinct aspects of barrier integrity. Moreover, systematic reviews such as Kurz *et al.* (2022) [16] and Huang *et al.* (2020) [17] integrate findings across methodologies and populations, providing broader context but with inherent heterogeneity. In summary, the present findings support a growing consensus that BBB and NVU dysfunction are central to Alzheimer's disease pathophysiology and correlate with cognitive impairment severity. Continued integration of neurovascular biomarkers into clinical research may enhance early detection and inform vascular targeted therapeutic strategies.

## Conclusion:

This study demonstrates those blood-brain barrier breakdown and neurovascular unit dysfunctions are significantly associated with Alzheimer's disease and correlate strongly with the severity of cognitive impairment. The findings suggest that cerebrovascular dysfunction is not merely a secondary consequence but a central contributor to disease progression. Impaired BBB integrity and reduced neurovascular coupling may exacerbate neuroinflammation, cerebral hypoperfusion and impaired waste clearance. These vascular abnormalities offer potential biomarkers for early detection of Alzheimer's disease. Targeting the neurovascular unit may therefore represent a promising therapeutic strategy to slow cognitive decline.

## Limitations:

This study has several limitations that should be considered when interpreting the findings. First, the cross-sectional design limits the ability to establish causal relationships between blood-brain barrier breakdown, neurovascular unit dysfunction and cognitive decline in Alzheimer's disease, as temporal progression could not be assessed. Second, the relatively modest sample size of 100 participants from a single center may restrict the generalizability of the results to broader and more diverse populations. Third, although multiple biomarkers and imaging parameters were used to assess BBB and NVU integrity, invasive cerebrospinal fluid markers and advanced molecular imaging were not uniformly available for all participants, which may have limited the sensitivity of barrier dysfunction detection. Fourth, potential residual confounding from unmeasured factors such as genetic predisposition, lifestyle variables and medication effects cannot be completely excluded despite statistical adjustment for major vascular risk factors. Finally, the study did not include longitudinal follow-up to evaluate how BBB and NVU changes evolve over time or predict conversion from early cognitive impairment to Alzheimer's disease, highlighting the need for future prospective studies.
